# Direct Identification of Insulator Components by Insertional Chromatin Immunoprecipitation

**DOI:** 10.1371/journal.pone.0026109

**Published:** 2011-10-17

**Authors:** Toshitsugu Fujita, Hodaka Fujii

**Affiliations:** Combined Program on Microbiology and Immunology, Research Institute for Microbial Diseases, Osaka University, Osaka, Japan; National Institutes of Health, United States of America

## Abstract

Comprehensive understanding of mechanisms of epigenetic regulation requires identification of molecules bound to genomic regions of interest *in vivo*. However, non-biased methods to identify molecules bound to specific genomic loci *in vivo* are limited. Here, we applied insertional chromatin immunoprecipitation (iChIP) to direct identification of components of insulator complexes, which function as boundaries of chromatin domain. We found that the chicken β-globin HS4 (cHS4) insulator complex contains an RNA helicase protein, p68/DDX5; an RNA species, steroid receptor RNA activator 1; and a nuclear matrix protein, Matrin-3, *in vivo*. Binding of p68 and Matrin-3 to the cHS4 insulator core sequence was mediated by CCCTC-binding factor (CTCF). Thus, our results showed that it is feasible to directly identify proteins and RNA bound to a specific genomic region *in vivo* by using iChIP.

## Introduction

Epigenetic regulation of eukaryotic genomic regions is mediated by molecular complexes in the context of chromatin [1]. However, biochemical nature of chromatin domains is poorly understood, mainly because methods for biochemical and molecular biological analysis of chromatin structure are limited [2]–[7]. In this regard, it was recently reported that proteomics of isolated chromatin segments (PICh) using specific nucleic acid probes can be used to identify components of telomere complexes retaining multiple (10^3^–10^4^) DNA repeats [8]. However, methods to directly identify proteins bound to low copy number genes have not been reported.

Insulators function as boundaries of chromatin domain. The genes flanked by insulators are protected from inappropriate trans-elements outside of the insulators as well as chromatin silencing [9], [10]. Regulators of insulator function have been extensively analyzed. For example, it has been shown that CCCTC-binding factor (CTCF) binds to insulator DNA and plays a critical role in insulator function [11], [12]. Other insulator-associated molecules have also been identified [9], [10], [13]. However, precise molecular mechanisms of insulator function are yet to be elucidated.

We recently developed insertional chromatin immunoprecipitation (iChIP), which is a method to biochemically isolate a genomic region of interest [14]. Here, we applied iChIP to direct identification of components of the chicken insulator HS4 (cHS4), which regulates expression of β-globin genes [15]. By using iChIP, we found that the cHS4 insulator complex contains an RNA helicase protein, p68/DDX5; an RNA species, steroid receptor RNA activator 1 (SRA1); and a nuclear matrix protein, Matrin-3, *in vivo*. In addition, we showed that binding of p68 and Matrin-3 to the cHS4 insulator core sequence (cHS4-core) is mediated by CTCF. Thus, our results showed that it is feasible to directly identify proteins and RNA bound to a specific genomic region *in vivo* by using iChIP.

## Results and Discussion

### Direct isolation of insulator protein components, p68 and Matrin-3, by iChIP-mass spectrometry

**Figure 1:**
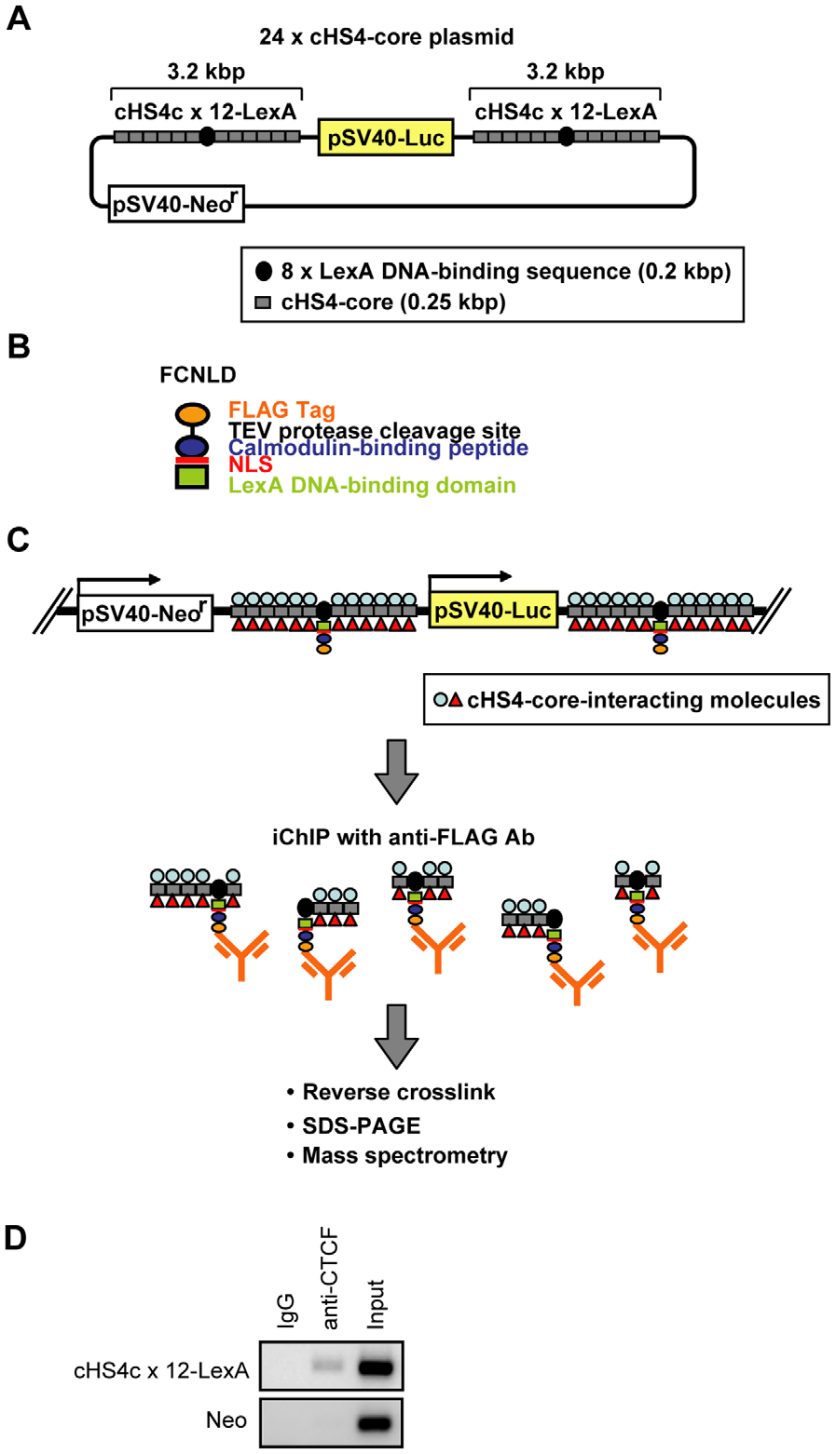
Scheme of identification of insulator components by iChIP. (**A**) The 24×cHS4-core plasmid, which possesses 2 copies of the cHS4c×12-LexA cassettes. (**B**) FCNLD protein consisting of 2× FLAG-tag, a TEV protease cleavage site, the calmodulin-binding peptide, the nuclear localization signal (NLS) of SV40 T-antigen and the DNA-binding domain of LexA. (**C**) The 24×cHS4-core plasmid was randomly integrated into the genome of a FCNLD-expressing Ba/F3-derived cell line. To isolate the cHS4-core region and associated molecules, the fixed chromatin extracted from the FCNLD/cHS4-core cells was fragmented by sonication and subjected to immunoprecipitation with anti-FLAG Ab. The isolated chromatin complexes were reverse-crosslinked and subjected to SDS-PAGE, silver-staining, followed by mass spectrometry. (**D**) Interaction of CTCF with the cHS4-core transgene *in vivo* shown by ChIP assay with anti-CTCF Ab.

We applied iChIP [14] to non-biased search for proteins interacting with cHS4-core *in vivo* as shown in Figure 1. We constructed the 24×cHS4-core plasmid possessing two copies of the cHS4c×12-LexA cassette, in which 8× repeats of the LexA binding sequence was flanked at each side by six copies of the cHS4-core sequence (Figure 1A). It was shown that transfected 250-bp cHS4-core sequences retain insulator activity [15], [16]. The 24×cHS4-core plasmid or negative control plasmid (pGL3C-Neo) was transfected into a mouse hematopoietic Ba/F3-derived cell line [17] expressing the FCNLD protein consisting of 2× FLAG-tag, the calmodulin-binding peptide, the nuclear localization signal (NLS) of SV40 T-antigen, and the DNA-binding domain of LexA (Figure 1B) [14]. The resultant FCNLD/cHS4-core cell line had one copy of the 24×cHS4-core plasmid integration in its genome (Figure S1). ChIP assay with antibody (Ab) against CTCF showed specific interaction of CTCF with the exogenous cHS4-core in the genome of the FCNLD/cHS4-core cell line *in vivo* (Figure 1D), indicating that the exogenous cHS4-core retains the ability to recruit insulator components.

**Figure 2:**
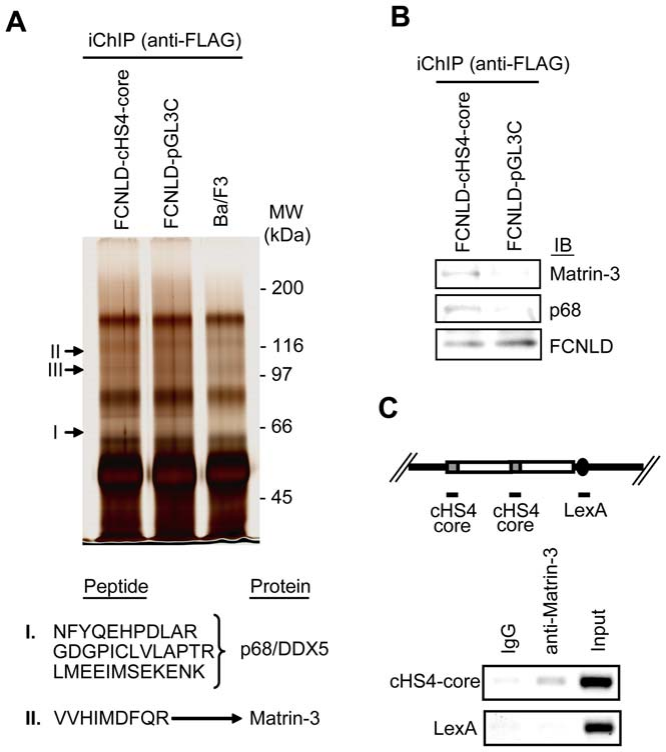
Direct identification of protein components of the cHS4 insulator complex by iChIP-mass spectrometry. (**A**) Identification of insulator-binding proteins. Upper panel: Proteins isolated by iChIP were subjected to SDS-PAGE followed by silver-staining. Enlarged images of bands specific to the FCNLD/cHS4-core cell line are shown in Figure S2. Lower panel: The protein bands were subjected to mass spectrometry (LC-MS/MS) and identified as p68/DDX5 (I) and Matrin-3 (II). MW: molecular weight. See Figure S3 for MS/MS data. (**B**) Detection of p68 and Matrin-3 by iChIP followed by immunoblot analysis with anti-p68 and anti-Matrin-3 Ab. (**C**) Interaction of Matrin-3 with cHS4-core region *in vivo*. Upper panel: The plasmid possessing 2 copies of the cHS4 insulator and 8× repeats of the LexA binding sequence was randomly integrated in the genome of Ba/F3 cells. Lower panel: Specific interaction of Matrin-3 with cHS4-core *in vivo*. ChIP assay with anti-Matrin-3 Ab was performed to detect Matrin-3 binding.

4×10^7^ of FCNLD/cHS4-core, FCNLD/pGL3C, and parental Ba/F3 cell lines were subjected to crosslinking with formaldehyde, sonication, and immunoprecipitation with anti-FLAG Ab. After direct reverse crosslinking in SDS sample buffer, the immunoprecipitated complexes were resolved by 7% SDS-PAGE and subjected to silver-staining. Three bands were specifically detected in the immunoprecipitants from the FCNLD/cHS4-core cell line (Figure 2A and Figure S2). These bands were excised and subjected to liquid chromatography coupled with tandem mass spectrometry (LC-MS/MS) to reveal that the band I corresponds to an RNA helicase, p68/DDX5 [18], whereas the band II is a nuclear matrix protein, Matrin-3 (Figure 2A and Figure S3) [19]. LC-MS/MS failed to identify the band III.

We failed to identify CTCF by iChIP-mass spectrometry. This is presumably because CTCF (molecular weight (MW) 130–150 kDa) was concealed by non-specific bands whose MWs are 130–150 kDa (Figure 2A). We also failed to identify Nucleophosmin/B23, which has been shown to be a component of cHS4 [20]. It is likely that Nucleophosmin/B23 (MW 37–40 kDa) was covered by the heavy chain of anti-FLAG Ab, which was directly denatured in the SDS sample buffer and subjected to SDS-PAGE and silver-staining in this study.

iChIP followed by immunoblot analysis with anti-p68 and anti-Matrin-3 Ab confirmed that the immunoprecipitated complexes from the FCNLD/cHS4-core cell line contains p68 and Matrin-3 (Figure 2B). It has been reported that p68 is one of components of the *gypsy* insulator of *D. melanogaster* [21]. A recent report also showed interaction of p68 with mammalian insulator DNA elements via CTCF [22]. In contrast, involvement of Matrin-3 in insulator function has not been reported. ChIP assay confirmed that Matrin-3 is specifically associated with the cHS4 insulator transgene *in vivo* (Figure 2C).

### p68 and Matrin-3 interact indirectly with cHS4-core

**Figure 3:**
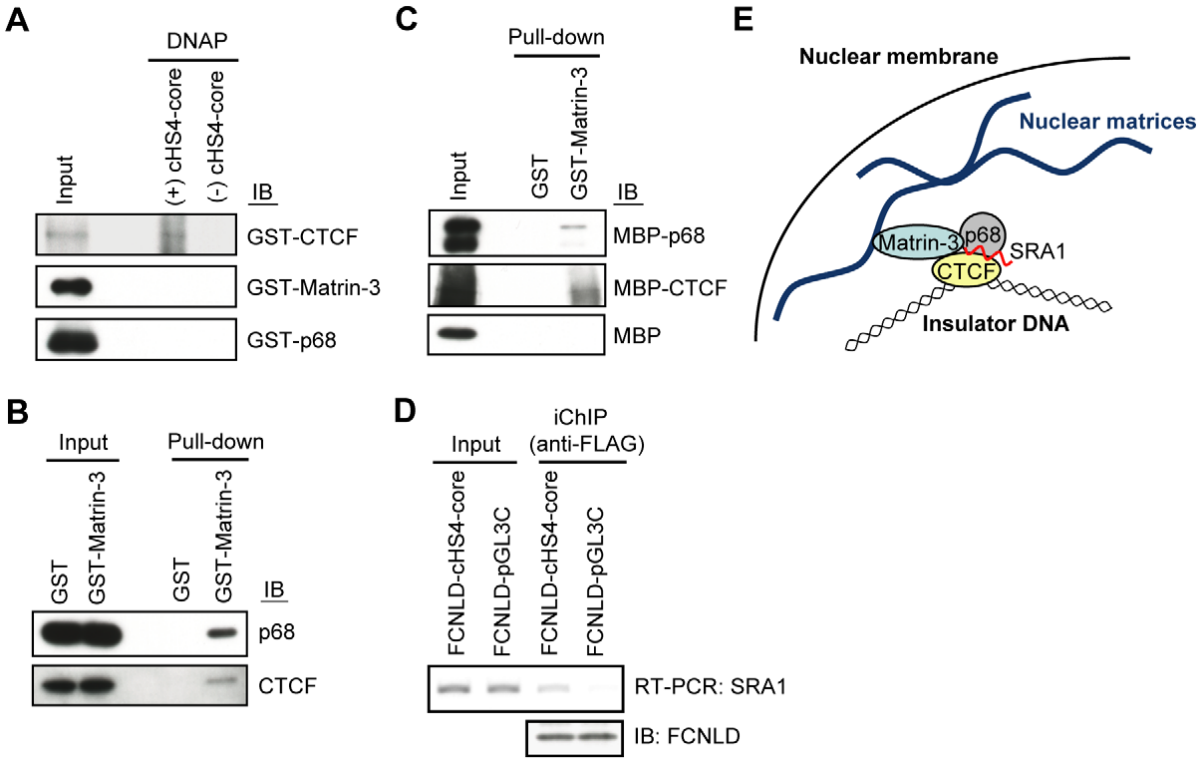
The mode of interaction of insulator components. (**A**) GST-fused CTCF directly binds to the cHS4-core DNA, whereas GST-Matrin-3 or -p68 failed to show direct interaction with the cHS4-core DNA. DNAP assay was performed with GST-fused proteins and the biotinylated cHS4-core DNA. Immunoblot analysis was performed with anti-CTCF, anti-Matrin-3, or anti-p68 Ab. (**B**) Interaction of Matrin-3 with p68 and CTCF. Pull-down assay was performed with GST-Matrin-3 and Ba/F3 nuclear extracts. Immunoblot analysis was performed with anti-p68 or anti-CTCF Ab. (**C**) Matrin-3 directly interacts with p68 and CTCF. Pull-down assay was performed with GST-Matrin-3, MBP-p68, MBP-CTCF, and MBP. Immunoblot analysis was performed with anti-MBP Ab. (**D**) Detection of SRA1 in the cHS4-core complex *in vivo* by iChIP-RT-PCR. (**E**) A nuclear matrix protein, Matrin-3, tethers the HS4 insulator to the nuclear matrix via CTCF and SRA1-associated p68.

Next, we examined potential direct interaction of cHS4-core with Matrin-3 as well as p68 using DNA-affinity precipitation assay (DNAP) [23]. The cHS4-core DNA conjugated with beads were incubated with recombinant glutathione S-transferase (GST)-fused proteins. Immunoblot analysis showed that cHS4-core directly interacts with GST-CTCF (Figure 3A) as shown previously [11]. In contrast, GST-p68 did not interact with cHS4-core directly (Figure 3A), consistent with the previous report that p68 interacts with insulator DNA via CTCF [22]. In this regard, GST-Matrin-3 failed to interact with cHS4-core (Figure 3A), suggesting that interaction of Matrin-3 with the cHS4-core DNA is indirect.

To elucidate the mode of interaction of Matrin-3 with cHS4-core, we subsequently examined binding of Matrin-3 to CTCF and p68 using GST pull-down assay. GST or GST-Matrin-3 was incubated with nuclear extracts of Ba/F3 cells. Immunoblot analysis showed that Matrin-3 interacts with p68 and CTCF (Figure 3B). Next, we examined direct interaction of Matrin-3 with p68 and/or CTCF. As shown in Figure 3C, GST-Matrin-3 interacted directly with both maltose-binding protein (MBP)-fused p68 and CTCF. These results indicate that insulator DNA interacts with Matrin-3 via p68 and CTCF. It has been suggested that tethering of insulators to the nuclear matrix, inner membranes of nuclei, or outer membranes of nuclear components is necessary for their function [20], [24]–[26]. The flanking insulators interact each other to establish an insulator-loop, isolating the flanked genes [9], [10]. It is possible that Matrin-3 may play some roles in tethering of insulator complexes to the nuclear matrix (Figure 3E). Elucidation of the role of Matrin-3 in the insulator function is an important future issue.

### Direct isolation of an RNA component of insulator complexes by iChIP

Recently, it was reported that p68 binds to CTCF in the presence of an RNA species, SRA1 [22]. We examined whether direct detection of RNA is feasible by using iChIP. As shown in Figure 3D, iChIP combined with RT-PCR revealed specific interaction of SRA1 with cHS4-core in the genome *in vivo*, showing that iChIP enables us to directly identify RNA species associated with genomic regions of interest.

### Conclusions

In this study, we applied iChIP to identification of molecules bound to the cHS4 insulator *in vivo*. It is noteworthy that only 4×10^7^ cells containing 24 copies of cHS4-core per genome are sufficient to identify p68 and Matrin-3 as protein components of the insulator complex (Figure 1 and Figure 2), showing that iChIP-mass spectrometry enables us to directly identify proteins bound to low copy number genes *in vivo*. In addition, we showed that direct identification of an RNA component of insulator complexes is feasible by iChIP (Figure 3D). Recently, involvement of non-coding RNA in various epigenetic mechanisms is suggested [27]. iChIP combined with DNA microarray analysis or next generation sequencing can be used for comprehensive analysis of RNA interacting with a target genomic region of interest. Thus, our results showed that it is feasible to directly identify proteins and RNA bound to a specific genomic region *in vivo* by using iChIP.

## Materials and Methods

### Plasmid construction

To construct pGL3C-Neo, the neomycin resistance gene in pcDNA3.1-myc.His.A (Invitrogen) was amplified by PCR and inserted at *Nhe* I/*Sma* I site of pGL3-Control (Promega). cHS4-core was amplified by PCR and cloned in pMD20-T vector (TaKaRa). Six copies of the core sequence (6×cHS4-core) were tandemly linked on the vector. The 8× repeats of the LexA binding sequence [28] were flanked by the 6×cHS4-core at each side to generate the cHS4c×12-LexA cassette. The cHS4c×12-LexA cassette was inserted into both the *Xho* I and *Sal* I sites of pGL3C-Neo to generate pGL3C-Neo-cHS4c×24-LexA×2. To construct pGL3C-2×cHS4-LexA, 2×cHS4 from pUC-2×cHS4 [29] and the 8× repeats of the LexA binding sequence was inserted into the *Xho* I site of pGL3C-Neo.

### Cell lines

Ba/F3 [17] and FCNLD-expressing Ba/F3-derived cells [14] were grown as described previously [30]. An FCNLD-expressing Ba/F3-derived cell line (1×10^7^ cells) was transfected with *Kpn* I-digested pGL3C-Neo-cHS4c×24-LexA×2 (10 µg) or *Kpn* I-digested pGL3C-Neo (10 µg) by electroporation using Gene Pulser II (Bio-Rad) at 250 V, 975 µF. The transfected cells were selected in the presence of G418 (2 mg/ml) to establish the FCNLD/cHS4-core or the FCNLD/pGL3C cell line. Ba/F3 cells (1×10^7^ cells) were transfected with *Kpn* I-digested pGL3C-2×cHS4-LexA (10 µg) and selected to establish the cHS4×2-LexA cell line.

### Chromatin preparation

Cells (4×10^7^) were fixed with 1% formaldehyde at 37°C for 5 min. The chromatin fraction was extracted and fragmented by sonication as described previously [31] except for using 1.6 ml of Sonication Buffer (10 mM Tris pH 8.0, 150 mM NaCl, 1 mM EDTA, 0.5 mM EGTA, 0.1% sodium deoxycholate, 0.1% SDS, 40 U/ml recombinant RNase Inhibitor (Clontech) and UD-201 ultrasonic disruptor (TOMY SEIKO). The average length of chromatin fragments was about 2 kb.

### iChIP

The sonicated chromatin in Sonication Buffer was pre-cleared with 20 µg of normal mouse IgG (Santa Cruz Biotechnology) conjugated to 200 µl of Dynabeads-Protein G (Invitrogen) and subsequently incubated with 20 µg of anti-DYKDDDDK tag (anti-FLAG) Ab (WAKO) conjugated to 200 µl of Dynabeads-Protein G at 4°C for 20 h. The Dynabeads were washed one time each with 1 ml of Low Salt Wash Buffer (20 mM Tris pH 8.0, 150 mM NaCl, 2 mM EDTA, 1% Triton X-100, 0.1% SDS), High Salt Wash Buffer (20 mM Tris pH 8.0, 500 mM NaCl, 2 mM EDTA, 1% Triton X-100, 0.1% SDS), LiCl Wash Buffer (10 mM Tris pH 8.0, 250 mM LiCl, 1 mM EDTA, 0.5% IGEPAL-CA630, 0.5% sodium deoxycholate), and TE Buffer (10 mM Tris pH 8.0, 1 mM EDTA). The immunoprecipitants were incubated at 70°C for 10 min in 50 µl of 2× Sample Buffer (125 mM Tris pH 6.8, 10% 2-mercaptoethanol, 4% SDS, 10% sucrose, 0.004% bromophenol blue) to be eluted from the Dynabeads. The supernatant was incubated at 98°C for 20 min for reverse crosslinking and subjected to SDS-PAGE. The proteins were visualized by silver staining with Dodeca silver staining kit (Bio-Rad). Protein bands were excised and analyzed by mass spectrometry (LC-MS/MS) at DNA-chip Development Center for Infectious Diseases (RIMD, Osaka University). For iChIP-immunoblot analysis, immunoprecipitants were subjected to SDS-PAGE followed by immunoblot analysis with anti-p68 Ab (H-144, Santa Cruz Biotechnology), anti-Matrin-3 Ab (2539C3a, Santa Cruz Biotechnology), or Ab against the LexA DNA-binding domain (06-719, Millipore). For detection of RNA, iChIP was performed as described above except that 5 U/ml of recombinant RNase Inhibitor was added in the wash buffer. The immunoprecipitants were treated with 200 µg of Proteinase K at 65°C for 1 h in 300 µl of RNA Reverse Crosslinking Buffer (10 mM Tris pH 8.0, 1 mM EDTA, 1% SDS, 200 mM NaCl). After reverse crosslinking, total RNA was extracted with Isogen II RNA extraction kit (WAKO) and reverse-transcribed with ReverTra Ace qPCR RT kit (TOYOBO). The synthesized cDNA was used for PCR with AmpliTaq Gold 360 Master Mix (Applied Biosystems). PCR cycles were as follows: denaturing at 95°C for 10 min; 30 cycles of 95°C for 30 sec, 55°C for 30 sec, 72°C for 30 sec; and the final extending at 72°C for 2 min. The primers used in this experiment are shown in Table S1.

### ChIP

The sonicated chromatin in Sonication Buffer was pre-cleared with 3 µg of normal rabbit IgG (Santa Cruz Biotechnology) or normal goat IgG (Santa Cruz Biotechnology) conjugated to 30 µl of Dynabeads-Protein G (Invitrogen) and subsequently incubated with 3 µg of anti-CTCF Ab (H280, Santa Cruz Biotechnology) or anti-Matrin-3 Ab (C-20, Santa Cruz Biotechnology) conjugated to 30 µl of Dynabeads-Protein G at 4°C for 20 h. After washing, the immunoprecipitants were treated with 2 µg of RNase A (Roche) in 300 µl of TE Buffer at 37°C for 1 h and subsequently with 200 µg of Proteinase K (Roche) and 0.5% SDS at 65°C for at least 4 h. The DNA purified by phenol-chloroform extraction and ethanol precipitation was used as a template for PCR with AmpliTaq Gold 360 Master Mix. PCR cycles were as follows: denaturing at 95°C for 10 min; 30–40 cycles of 95°C for 30 sec, 55°C for 30 sec, 72°C for 30 sec; and the final extending at 72°C for 2 min. The primers used in this experiment are shown in Table S1.

### Purification of recombinant proteins

cDNA encoding human CTCF (GenBank: NM_006565) was inserted downstream of the *GST* gene in pFN2A (Promega) to generate pFN2A-CTCF. cDNA encoding human Matrin-3 (GenBank: NM_199189) and human p68 (GenBank: NM_004396) were inserted downstream of the *GST* gene in pGEX-6P-1 (GE Healthcare) to generate pGEX-Matrin-3 and pGEX-p68, respectively. cDNA encoding human CTCF and human p68 were inserted downstream of the *MBP* gene in pMAL-x5c (New England Biolabs) to generate pMAL-CTCF and pMAL-p68, respectively. BL21-CodonPlus(DE3)-RIPL (Agilent Technologies) was transformed with each plasmid and cultured at 37°C for 4 h in the presence of 0.1 mM IPTG to induce expression of the recombinant proteins. The GST-fused and MBP-fused proteins were purified with Glutathione Sepharose 4B (GE Healthcare) and Amylose resin (New England Biolabs), respectively.

### DNAP assay

Purified GST-fused proteins (100 ng) were incubated with the biotinylated cHS4-core sequence (1 µg) and Poly dI-dC (15 µg) in 500 µl of DNAP Buffer (20 mM HEPES pH 7.5, 80 mM KCl, 1 mM MgCl_2_, 1 mM EDTA, 0.1% Triton X-100, 10% glycerol, 0.5 mM DTT) for 1 h at 4°C. Streptavidin-coated Dynabeads M-270 (30 µl, Invitrogen) was added into the reaction mixture and incubated for 1 h at 4°C. After washing with DNAP Buffer, the precipitants were subjected to SDS-PAGE and immunoblot analysis with anti-CTCF Ab, anti-p68 Ab, and anti-Matrin-3 Ab (2539C3a, Santa Cruz Biotechnology).

### Pull-down assay

Purified GST-fused proteins (50 ng) were incubated with Ba/F3 nuclear extracts (10 µg) or MBP-fused proteins (50 ng) in 500 µl of Interaction Buffer (20 mM Tris pH 8.0, 137 mM NaCl, 1.5 mM MgCl_2_, 1 mM EGTA, 0.1% Triton X-100, 10% glycerol) for 1.5 h at 4°C. Glutathione Sepharose 4B (30 µl, 50% slurry) (GE Healthcare) were added into the reaction mixture and incubated for 1.5 h at 4°C. After washing with Interaction Buffer, the precipitants were subjected to SDS-PAGE and immunoblot analysis with anti-CTCF, anti-p68 and anti-MBP Ab (New England Biolabs).

## Supporting Information

Figure S1 **Copy number of pGL3C-Neo-cHS4c×24-LexA×2 integrated in the genome of the FCNLD/cHS4-core cell line.** The copy number was determined by monitoring the amplification of the *luciferase* gene by using real-time PCR. The extracted genomic DNA (225 ng) was subjected to real-time PCR with SYBR *Premix Ex Taq* II (Tli RNaseH Plus, TaKaRa) using the Applied Biosystems 7900HT Fast Real-Time PCR System. PCR cycles were as follows: denaturing at 95°C for 30 sec; 40 cycles of 95°C for 5 sec and 60°C for 34 sec. The primers used in this experiment are shown in Table S1. The mean of two experiments is shown; the error bar represents the range.
(TIF)

Figure S2 **Enlarged photos of bands subjected to LC-MS/MS.**
(TIF)

Figure S3 **Identification of peptides by LC-MS/MS.** (**A–C**) Peptides identified from the band (I). (**D**) A peptide identified from the band (II).
(TIF)

Table S1 **Primers used in this study.**
(DOC)
