# Supplementary figures and images for: Direct Identification of Insulator Components by Insertional Chromatin Immunoprecipitation
Source: PLoS One. 2011 Oct 17;6(10):e26109. doi: 10.1371/journal.pone.0026109 (PMC3197142; doi:10.1371/journal.pone.0026109)

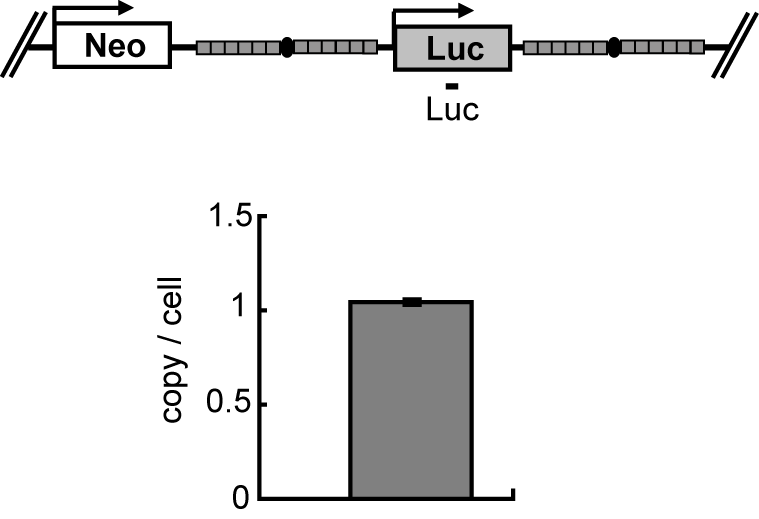

Supplement: Figure S1 — Copy number of pGL3C-Neo-cHS4c×24-LexA×2 integrated in the genome of the FCNLD/cHS4-core cell line. The copy number was determined by monitoring the amplification of the luciferase gene by using real-time PCR. The extracted genomic DNA (225 ng) was subjected to real-time PCR with SYBR Premix Ex Taq II (Tli RNaseH Plus, TaKaRa) using the Applied Biosystems 7900HT Fast Real-Time PCR System. PCR cycles were as follows: denaturing at 95°C for 30 sec; 40 cycles of 95°C for 5 sec and 60°C for 34 sec. The primers used in this experiment are shown in Table S1. The mean of two experiments is shown; the error bar represents the range. (TIF) [file pone.0026109.s001.tif]

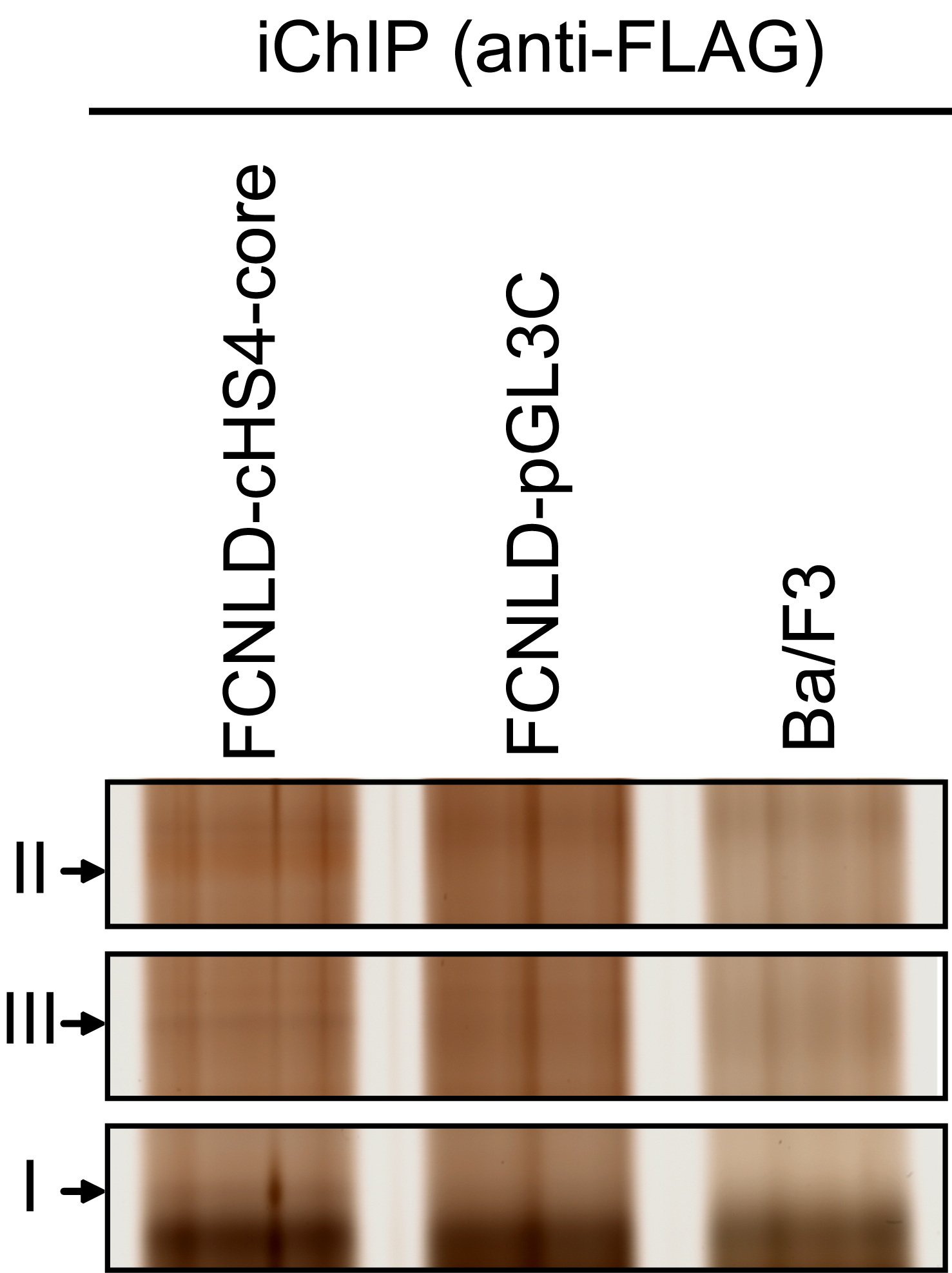

Supplement: Figure S2 — Enlarged photos of bands subjected to LC-MS/MS. (TIF) [file pone.0026109.s002.tif]

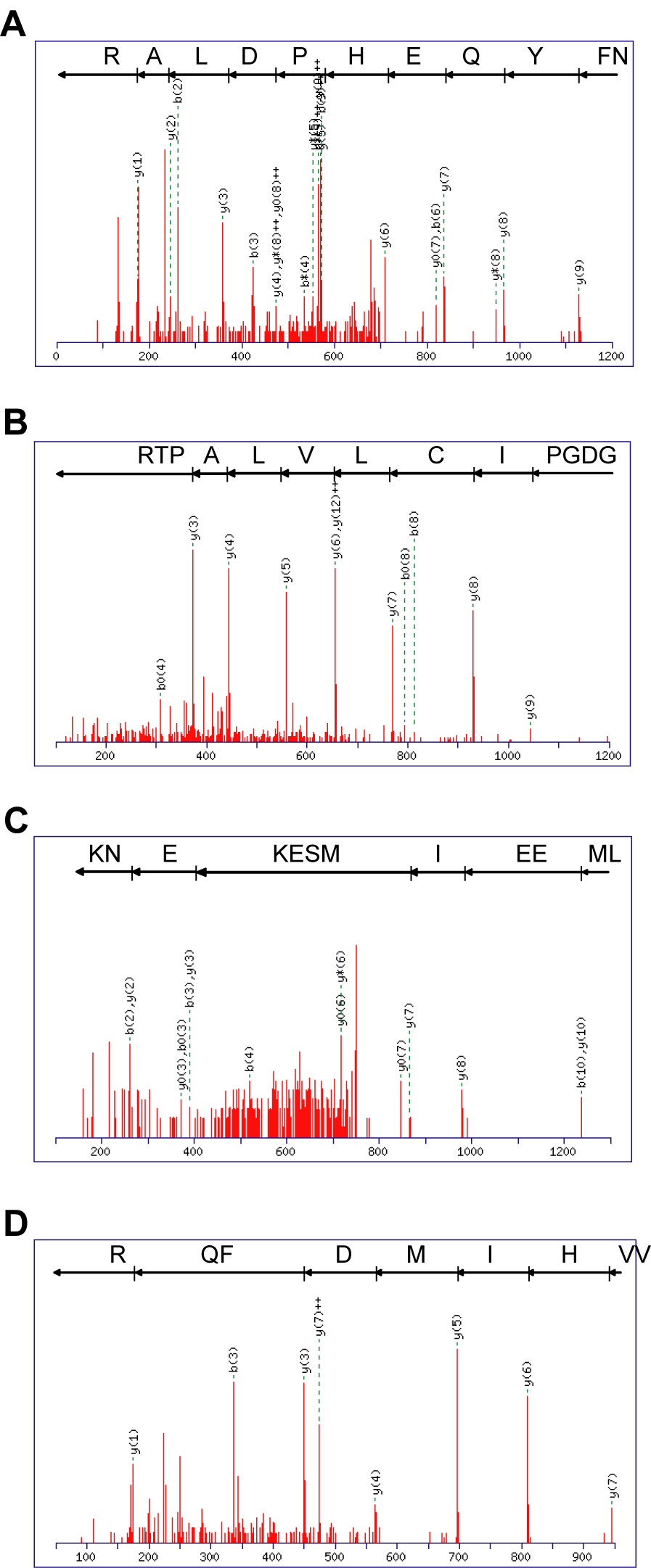

Supplement: Figure S3 — Identification of peptides by LC-MS/MS. (A–C) Peptides identified from the band (I). (D) A peptide identified from the band (II). (TIF) [file pone.0026109.s003.tif]
