# Supplementary material for: Direct Identification of Insulator Components by Insertional Chromatin Immunoprecipitation
Source: PLoS One. 2011 Oct 17;6(10):e26109. doi: 10.1371/journal.pone.0026109 (PMC3197142; doi:10.1371/journal.pone.0026109)
Supplement: Table S1 — Primers used in this study. (DOC) [file pone.0026109.s004.doc]

**Table S1**

| Name | Sequence (5' → 3') | Experiments |
| --- | --- | --- |
| LexA-F | TTCTCTATCGATAGGTACCTCG | Fig. 1D and Fig. 2C |
| LexA-R | TCTATTCAGCGGATCTCGAGCG | Fig. 1D and Fig. 2C |
| cHS4-core-F | ATTACGTCCCTCCCCCGCTA | Fig. 2C |
| cHS4-core-R | AGCTTTTTCCCCGTATCCCC | Fig. 2C |
| mSRA-F | agtggagatggcggagctgta | Fig. 3D |
| mSRA-R | gacctcagtcacatggtcaacc | Fig. 3D |
| Luc-F | TCAAAGAGGCGAACTGTGTG | Fig. S1 |
| Luc-R | GGTGTTGGAGCAAGATGGAT | Fig. S1 |

**Primers used in this study**
